# The Electric Shock during Acupuncture: A Normal Needling Sensation or a Warning Sign

**DOI:** 10.1155/2020/8834573

**Published:** 2020-11-02

**Authors:** Yongsong Guo, Ke Zhu, Jing Guo, Yongbing Kuang, Zhihui Zhao, Weihong Li

**Affiliations:** ^1^Basic Medical College, Chengdu University of Traditional Chinese Medicine, Chengdu 610075, China; ^2^Acupuncture College, Chengdu University of Traditional Chinese Medicine, Chengdu 610075, China; ^3^Department of Tuina, Hospital of Chengdu University of Traditional Chinese Medicine, Chengdu 610075, China; ^4^Department of Pneumology, Hospital of Chengdu University of Traditional Chinese Medicine, Chengdu 610075, China

## Abstract

The electric shock has been proposed as one of the new needling sensations in recent years. In acupuncture sensation scales, the electric shock is included by ASS and SNQS, but not SASS, MASS, and C-MMASS. Some scholars argue that the electric shock is a normal needling sensation, but some researchers do not agree with this view. This problem has not been resolved due to a lack of evidence from basic research. Literature and research point out that the electric shock is caused by inserting a needle into the nerve directly. A question of considerable scientific and practical interest is whether the electric shock should be a normal needling sensation. In this article, we review the historical documentation of the needling sensation and the process of formulating and improving acupuncture sensation scales to suggest that the electric shock may not be a normal needling sensation. Secondly, we collected and analyzed cases of nerve injury caused by acupuncture accompanied by the electric shock and why acupuncture caused the electric shock without nerve injury. It suggests that there may be a correlation between the electric shock and peripheral nerve injury, and acupuncture manipulation is an essential factor in adverse acupuncture events. Finally, we put forward that the electric shock during acupuncture is a warning sign that the peripheral nerve may be injured, rather than a normal needling sensation. In the future, we hope to have experimental studies on the mechanism of the electric shock or observational studies on the correlation between the electric shock and peripheral nerve injury to verify.

## 1. Introduction

The needling sensation refers to the subjective feeling that the recipient obtains in the acupoint after the acupuncturist inserts a needle into the acupoint or other acupuncture induction point and applies specific acupuncture techniques. The needling sensation is closely related to acupuncture treatment's therapeutic effect in traditional Chinese medicine [1–3]. Research has shown that the needling sensation may be an essential variable in acupuncture treatment studies' efficacy and mechanism [4]. The needling sensation can be briefly summarized as sourness, numbness, heaviness, or distension. The electric shock has been proposed as one of the new needling sensations in recent years. Shanghai and Sichuan Research Institute of Acupuncture and Meridian found in observational, experimental studies that acupuncture on nerves can produce the electric shock and proposed that the electric shock is a needling sensation [5, 6]. There is a special acupuncture treatment method called nerve trunk stimulation therapy in China, which uses the electric shock generated during the acupuncture process as the standard for Deqi. Some acupuncturists sum up their clinical acupuncture experience repeatedly and hold the same view [7–11].

However, other researchers suggested that the needle be stopped when the electric shock occurs during acupuncture to avoid nerve injury [12–14]. Patients provide the electric shock as a kind of needling sensation to the maker of acupuncture sensation scales. In the current representative acupuncture sensation scales, the electric shock is only included by ASS and SNQS. Now, due to the lack of basic research on the generation mechanism of the electric shock, we cannot answer positively whether the electric shock should be classified as a normal needling sensation. However, we discussed the historical literature records of needling sensation, the development process of acupuncture sensation scale, the adverse acupuncture events related to the electric shock, and nerve trunk stimulation therapy. And then, we put forward the point that there may be a correlation between the acupuncture and peripheral nerve injury. Acupuncture manipulation is an important influencing factor. Whether the electric shock is included in the scale needs more experimental evidence.

## 2. The Phenomenon of the Electric Shock

The electric shock appears on acupuncture sensation scales or needling sensation research, while we failed to find a specific description of the electric shock. Due to cultural and language background differences, there are other electric shock expressions, such as radiation and shock [15]. Therefore, based on relevant literature, we describe electric shock as an intense stimulation. It is produced by acupuncture into the human body at a certain depth or after lifting, twisting, and twirling manipulations, which can be instantaneously transmitted to the distal limbs. Within the stimulus conduction range, the limbs may appear numbness, severe pain, and other discomforts. Through summarizing the clinical literature on acupuncture, it is found that the electric shock only occurs when the needle penetrates the acupoint and reaches a certain depth [9, 10, 16].

Furthermore, according to the researcher's report [7, 17, 18], the electric shock will be quickly transmitted to the distal end of the limb, and its transmission range is consistent with the innervation area of the nerve trunk or branch around those acupoints. For instance, the researchers summarized the needling sensation of acupuncture at Huantiao (GB30). They pointed out that sciatic nerve trunks stabbed at different angles and depths will produce the electric shock, which can quickly be conducted to different innervation areas of the lower extremities [19].

## 3. Records Related to the Electric Shock in Acupuncture Ancient Literature

Acupuncture has a history of more than two thousand years in China, and all generations of acupuncture scholars have summarized the needling sensation. Since the ancients did not know about electricity, we took the radiation (the feeling of radiating to the far end in an instant) that must exist in the electric shock as the literature search object. However, we did not find any related records. On the contrary, in *The Miraculous Pivot*, the transmission phenomenon of needling sensation is described as “Under the acupuncture manipulation, the feeling of Deqi will be transmitted to the affected area like insects and ants crawling.” This conveys an important message to us that the transmission of needling sensation is slow and will not be transmitted to the far end instantly. *The Miraculous Pivot* records process of Deqi as “The feeling of Deqi during acupuncture is slow and comfortable.” In general, the needling sensation is slowly produced and gradually transmitted to the affected area under acupuncture manipulation. As *Plain Questions* mentioned, “Deqi needs to be obtained after correct acupuncture manipulation.” Besides, *The A-B Classic of Acupuncture and Moxibustion* records, “Febrile disease can be treated with acupuncture ST 43. Acupuncture ST 43 makes the feet feel cool through acupuncture manipulation. After the cooling sensation is transmitted from the foot to the knee joint, the needle is removed.” It is showed that the needling sensation is conducted to the affected area with acupoints as the center. This rule is also summarized in *Great Compendium of Acupuncture and Moxibustion* and *Willful Intercept of Acupuncture*.

On the contrary, the conduction direction of the electric shock is fixed. The electric shock is conducted from the location where the nerve is punctured to the distal limb. The electric shock does not conform to acupuncture scholars' summary of the content of needling sensation in the past dynasties. Ancient Chinese medical literature does not support that the electrical shock is a normal needling sensation.

## 4. Opinions of the Maker of Acupuncture Sensation Scales

The makers of needle-sense scales are divided on whether the needling sensation should include the electric shock. Under the background of the international popularization of acupuncture, it is necessary to establish objective and unified standards of the needling sensation to evaluate acupuncture research and treatment more scientific. The researchers have developed several representative acupuncture sensation scales, including acupuncture sensation scale (ASS) [20], the subjective acupuncture sensation scale (SASS) [21], MGH acupuncture sensation scale (MASS) [22], Southampton needle sensation questionnaire (SNSQ) [23], visual analog scales (VAS) [24], and the Modified MASS-Chinese version (C-MMASS) [25] (Table 1). The content of needling sensation comes from acupuncture experiments and needling sensation questionnaire research [26, 27].

ASS: acupuncture sensation scale; SASS: the subjective acupuncture sensation scale; MASS: MGH acupuncture sensation scale; SNSQ: Southampton needle sensation questionnaire; C-MMASS: the Modified MASS-Chinese version.

Vincent et al. [20] developed the ASS by providing a list of adjectives describing pain in the McGill Pain Questionnaire (MPQ) [28] to acupuncturists and patients for selection. Kong et al. [21] developed SASS through acupuncture experiments. The MASS was upgraded from the SASS and perfected by referring to the ASS. White et al. [23] developed the SNSQ after interviewing patients based on the existing scales and acupuncture experts' reviewing. Yu et al. [25] developed an acupuncture sensation scale suitable for the Chinese population through acupuncture experiments based on MASS. Examining the process of formulating and gradually improving these scales, we found that the electric shock was included in ASS and SNSQ but not in SASS, MASS, and C-MMASS. Furthermore, the electric shock is a weak factor in the ASS. SNSQ left the electric shock because some patients have experienced acupuncture treatment, and White et al. pointed out that the electric shock should be viewed as separate or individual items describing needle sensation in the SNSQ. The electric shock of ASS and SNQS mainly comes from experienced acupuncturists and patients' subjective feelings on needling sensation, and the maker cannot be sure whether the information obtained is correct. Based on the current information, whether the electric shock should be classified as a normal needling sensation requires basic research evidence to prove it.

## 5. Case Reports of Peripheral Nerve Injury Caused by Acupuncture with the Electric Shock

Reviewing the literature [29–31], adverse events of acupuncture mainly include internal organs, tissue, or nerve injury, especially for pneumothorax and central nervous system injury. We searched the following databases to find the case reports published from 1990 to 2019: VIP science and technology periodical database (CQVIP), China National Knowledge Infrastructure (CNKI), Wanfang Database (WF), and PubMed. Search terms included “acupuncture, electro-acupuncture” combined with “adverse event, adverse reaction, and peripheral nerve injury.” We found 11 documents related to peripheral nerve injury caused by acupuncture. After excluding needle breakage and unrecorded needling sensation, five documents are remaining related to the occurrence of the electric shock (or similar expression) during acupuncture. Those articles reported one case of median nerve injury caused by acupuncture at Neiguan (PC6) [32], three cases of common peroneal nerve injury caused by acupuncture at Yanglingquan (GB34) [33–35], and one case of sciatic nerve injury caused by acupuncture at Huantiao (GB30) [36]. We will briefly describe in Table 2.

In those adverse events, patients had different degrees of electric shock and other discomforts during acupuncture. The operator continued applying acupuncture while they did not realize that the electric shock would cause adverse consequences. In case one, case four, and case five, the operators believe that the electric shock is a strong reflection of Deqi. When the patient has the electric shock during acupuncture, the operator strengthens the electric shock's intensity through acupuncture manipulation. In the above cases, the conclusions from the neurological examination or electromyography examination suggested peripheral nerve injury. Doctors pointed out that it was caused by the peripheral nerve being stabbed by the needle.

## 6. The Electric Shock and Nerve Trunk Stimulation Therapy

In China, the direct acupuncture method, the nerve trunk to treat diseases, is called nerve trunk stimulation therapy. This therapy emerged in the 1970s and became popular in the 1980s to 1990s. It is a research result combining meridian and neuroanatomy. Traditional acupoints are interpreted as nerve trunk stimulation points [37, 38]. In the clinical application of nerve trunk stimulation therapy, the operator uses a needle to directly acupuncture the nerve trunk to cause the patient to generate the electric shock. In order to cause the patient to produce the electric shock, the operator will perform acupuncture manipulation for stimulating Qi of meridians at the corresponding nerve trunk stimulation point. Researchers using this therapy have given relevant points for attention. In the process of acupuncture, when the patient feels the electric shock, the needle should be slightly lifted to make the needle tip away from the nerve trunk; immediately afterward, other acupuncture operations were no longer used. This kind of electric shock will be converted to other needling sensation [39, 40]. The points for attention are also reflected in the xingnao kaiqiao acupuncture therapy by Professor Shi Xuemin to avoid nerve injury [12, 41–43]. Researchers of nerve trunk stimulation therapy and Professor Shi Xuemin emphasize that the electric shock should not be sought too much, as they think it may injure the nerves. These two acupuncture therapies make the needle tip to leave the nerve trunk after the electric shock is generated during acupuncture, and the basic acupuncture operation of lifting, twisting, and twirling is no longer applied to avoid repeated puncture of the nerve.

Streitberger et al. [44, 45] conducted an observational, experimental study of acupuncture. During the process of acupuncture P6 (pericardium 6) and puncturing the median nerve with the needle, it was found that the subject had already Deqi before the needle touched the median nerve (the recorded needling sensation did not contain the electric shock). In the experiment, the median nerve was punctured in 14 cases, abnormal sensations were reported in 10 cases, radioactive needling sensation was reported in 8 cases, and the needle was immediately removed due to intractable electric pain in 1 case. The experimental study results suggest no connection between Deqi and acupuncture nerves, but it is necessary to avoid puncturing nerves through relevant training. Although the electric shock occurred during the experiment, a follow-up one week later showed no nerve injury in the subjects whose nerves were punctured. A peripheral nerve consists of 50% neurons, 50% fat, and connective tissue. The experiment did not use acupuncture manipulation to repeatedly puncture the nerve, reducing the possibility of the needle puncturing the nerve tract. The possibility of nerve injury is low in a single nerve puncture accompanied by the electric shock, but the probability will increase if the nerve is punctured multiple times.

## 7. The Possible Consequences of Repeated Puncturing Nerves by Acupuncture Manipulation

**Figure 1:**
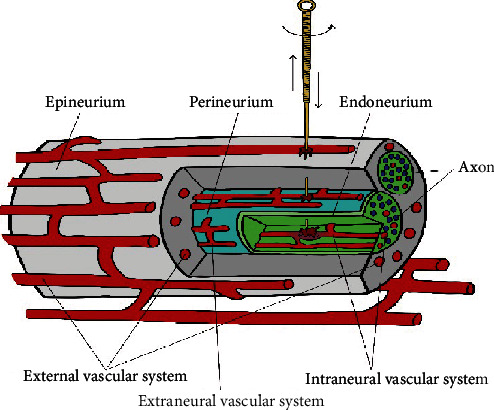
Microvascular injury on peripheral nerves.

**Figure 2:**
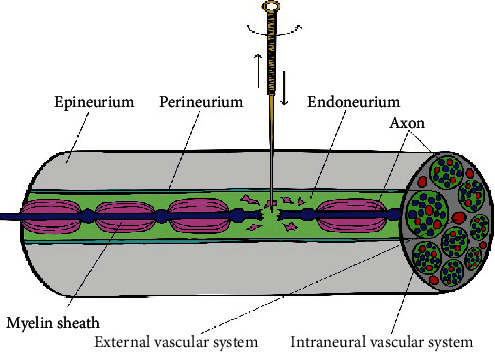
Traumatic demyelination and axonotmesis about peripheral nerve.

Repeatedly piercing the nerve in seeking the Electric Shock by lifting, twisting, and twirling is crucial to nerve injury. At present, there is no basic experimental study on puncturing nerves with acupuncture needles. Therefore, we refer to the situation of nerve injury caused by injection needles to explore the possible injury caused by acupuncture repeatedly puncturing the nerve. The nerve is regarded as a unique organ composed of nerve tissue, connective tissue, and microvasculature. There is no significant difference in the nerve's local inflammation caused by needles of different diameters [46, 47]. Both extraneural and intraneural vascular systems are likely to be punctured when needles stab the nerve. In particular, extraneural vascular has more opportunities of being injured than intraneural vascular [48, 49] (Figure 1). Acupuncture may also cause nerve fiber injury during twirling, lifting, and thrusting (Figure 2).

During twirling, lifting, and thrusting, the extraneural and intraneural vascular system nerve may be punctured. Moreover, the hematoma can cause inflammation and squeeze the surrounding nerve tissue.

During twirling, lifting, and thrusting, myelin sheath and axon may be destroyed, and then, the injury triggers classic inflammation and neuropathic inflammation.

## 8. Summary

By reviewing ancient Chinese medicine books, the formulation process of acupuncture sensation scale, adverse events of acupuncture, and the related literature of nerve stem stimulation therapy, our view is that the electric shock in the acupuncture process is a warning sign to indicate that the peripheral nerve may have been injured. Furthermore, acupuncture manipulation is an essential factor in causing nerve injury. The reason is as follows: no record of the needling sensation similar to the electric shock is found in ancient Chinese medicine books; the electric shock is inconsistent with the documented needling sensation in the conduction speed and direction; the scale makers that included the electric shock into the acupuncture sensation scales have doubts about whether the electric shock is a normal needling sensation; moreover, the electric shock is not derived from acupuncture experiments, but from the self-summary of acupuncturists and patients. Analyzing the case of nerve injury caused by acupuncture accompanied by the electric shock and comparing it with the nerve stem stimulation therapy in which the electric shock appears but there are no acupuncture accidents have been reported. We propose that obtaining electrical shock through acupuncture manipulation during acupuncture may cause nerve injury. However, the current studies supporting that the sense of electric shock belongs to normal needle sensation or that the sense of electric shock indicates nerve injury are limited to expert experience and adverse acupuncture case reports. In the future, it is necessary to conduct clinical observational studies or experimental studies on the correlation between the electric shock and nerve injury.

## Figures and Tables

**Table 1:** The contents of representative acupuncture sensation scales.

Group	Scale	Year	Content about needling sensation
Vincent et al. [20]	ASS	1989	Dull-heavy sensations, a general intensity dimension, spreading, stinging, hot, sharp, and electric sensations
Kong et al. [21]	SASS	2005	Heaviness, stabbing, tingling, throbbing, burning, fullness, numbness, soreness, and aching
Kong et al. [22]	MASS	2007	Heaviness, deep pressure, soreness, aching, sharp pain, warmth, cold, fullness/distension, tingling, numbness, dull pain, and throbbing
White et al. [23]	SNSQ	2008	Heavy, pressure, spreading, stinging, tingling, deep ache, dull ache, warm, uncomfortable, bruised, fading, numb, twinge, throbbing, pricking, and electric shock
Yu et al. [25]	C-MMASS	2012	Soreness, aching, pressure, heaviness, fullness, tingling numbness, sharp pain, dull pain, warmth, cold, and throbbing

**Table 2:** Peripheral nerve injury associated with acupuncture.

Case	Acupoint	Patient sensation during acupuncture	Patients sensation or symptoms after acupuncture	Result of examine	Damaged nerve
(1) Wang [32]	Neiguan (PC6)	Electric shock, burning and radiated to the hand	The thumb, index finger, and middle finger could not flex and had difficulty in straightening	The affected limb has limited movement, skin temperature, thermesthesia, and pain are weaker than the healthy side	Median nerve
(2) Sobel et al. [33]	Yanglingquan (GB34)	Pain and radiated to the right lower limb	Burning, pain, unable to move the right foot	The extensor hallucis longus, extensor digitorum longus, and peroneal muscles were all graded as 2/5, and the anterior tibialis muscle was graded as 4/5	Peroneal nerve
(3) Sato et al. [34]	Yanglingquan (GB34)	Pain and radiated to the left leg	Pain, burning, numbness, and weakness in the left leg	EMG: compound muscle action potentials of the peroneal nerve in the left leg showed a remarkable decrease in amplitude distal to the level of the fibular head	Peroneal nerve
(4) Ruan et al. [35]	Yanglingquan (GB34)	Electric shock, pain, and radiated to the left lower leg	Pain, burning in the calf to the back of the foot, weakness in the lower extremities	EMG examination revealed injury of the common peroneal nerve	Peroneal nerve
(5) Wang [36]	Huantiao (GB30)	Electric shock, pain, and radiated down to the right foot	Numbness and weakness in the right leg	No record	Sciatic nerve

EMG: electromyogram.

## Data Availability

The data supporting this systematic review are from previously reported studies and datasets, which have been cited. The picture used to support this systematic review are included within the article.
